# Real‐world clinical outcomes of patients with high‐risk endometrial cancer or endometrial carcinosarcoma in England: A retrospective cohort study

**DOI:** 10.1111/jog.70042

**Published:** 2025-09-19

**Authors:** Vimalanand S. Prabhu, Erik Landfeldt, Eleanor Ralphs, Cheryl Teoh, Jess Ridsdale‐Smith, Karen Macey, Nikolay Trankov, Alexandrina Lambova, Jasmine Lichfield, Gemma Eminowicz

**Affiliations:** ^1^ Merck & Co., Inc. Rahway New Jersey USA; ^2^ IQVIA Stockholm Sweden; ^3^ IQVIA Ltd. Real World Solutions London UK; ^4^ MSD (UK) Limited London UK; ^5^ IQVIA Ltd. Real World Solutions Sofia Bulgaria; ^6^ Department of Oncology University College London Hospitals NHS Foundation Trust London UK

**Keywords:** disease‐free survival, endometrial neoplasms, retrospective studies, survival rate, treatment outcome

## Abstract

**Aim:**

The objectives of this study were to describe patient characteristics and estimate real‐world disease‐free survival (rwDFS) and overall survival (OS) from initiation of first adjuvant therapy among patients with high‐risk endometrial cancer (EC) in England.

**Methods:**

This was a retrospective cohort study based on data from Public Health England's National Cancer Registration and Analysis Service (NCRAS) between 2014 and 2023. Adult women with EC or endometrial carcinosarcoma at high risk of recurrence who received adjuvant therapy within 90 days after surgery were eligible for inclusion. We operationalized rwDFS as time to next treatment or death.

**Results:**

In total, 6036 women (mean age: 67 years; 86% White) were eligible for inclusion, with a mean follow‐up of 48 months. During the study period, 45% of patients experienced recurrence and 39% of patients died due to any cause. Median rwDFS and OS from initiation of adjuvant therapy were estimated at 4.56 years (95% CI: 4.14–5.12) and 8.85 years (95% CI: 8.15–9.82), respectively. Estimated 2‐year and 5‐year probabilities were 0.64 (95% CI: 0.63–0.65) and 0.49 (95% CI: 0.48–0.50) for rwDFS, and 0.78 (95% CI: 0.77–0.79) and 0.60 (95% CI: 0.58–0.61) for OS, respectively. Disease recurrence was associated with a 3.23‐fold higher risk of death (*p* < 0.001). Kendall's *τ* correlation coefficient between rwDFS and OS was 0.75 (95% CI: 0.69–0.80, *p* < 0.001).

**Conclusions:**

The results from our study underscore the substantial clinical burden and unmet medical need of women with high‐risk EC and the validity of rwDFS as a surrogate for OS.

## INTRODUCTION

Endometrial cancer (EC) is the most common gynecologic malignancy in Europe, with an age‐standardized incidence of about 20 per 100 000 women.^1^ Despite improvements in standards of care, approximately 18% of patients with EC experience recurrence within the first 2 years after their initial surgery.^2^ Recurrence likelihood is influenced by clinicopathological factors and tumor molecular profiles (e.g., mismatch repair [MMR] status or tumor protein TP53^3^, ^4^, ^5^, ^6^). Additionally, women with high‐risk EC—defined per the 2022 European Society for Medical Oncology (ESMO) guidelines as International Federation of Gynecology and Obstetrics (FIGO) stage I or II with myometrial invasion of non‐endometrioid histology or any histology with p53 mutation or FIGO stage III/ IVA EC^7^—have been reported to have up to four times higher recurrence rates,^8^, ^9^, ^10^ with increased mortality after recurrence.^9^, ^10^, ^11^

Patients with high‐risk EC are usually treated with curative intent surgery, including hysterectomy with bilateral salpingo‐oophorectomy, followed by adjuvant therapy, such as systemic chemotherapy with or without radiotherapy.^4^ A limited number of trials have investigated clinical outcomes in patients with high‐risk EC treated with different combinations of adjuvant treatment following surgery with curative intent. For example, the PORTEC‐3 trial investigated combined adjuvant chemotherapy and radiotherapy versus pelvic radiotherapy alone for women with high‐risk EC, and reported a benefit in 5‐year overall survival (OS) and failure‐free survival for the former treatment arm.^12^ However, the GOG 258 trial did not report benefits in relapse‐free survival for adjuvant chemotherapy with radiation than chemotherapy alone.^13^ More recently, immune checkpoint inhibitors (ICI)—previously limited to second line in advanced or recurrent EC following prior treatment with a platinum‐containing regimen—have been investigated in the adjuvant setting for patients with high‐risk EC. For example, the KEYNOTE‐B21 phase III trial evaluated the addition of pembrolizumab to adjuvant standard of care (SOC) chemotherapy (i.e., carboplatin + paclitaxel with optional radiation). While the addition of pembrolizumab did not meet its primary endpoint of improvement in disease‐free survival (DFS) in the entire population, a post‐hoc analysis demonstrated a clinically meaningful DFS improvement for patients with mismatch repair‐deficient (dMMR) tumors.^14^ Ongoing phase III NRG‐GY020 and ENGOT‐en14/RAINBO MMRd‐GREEN trials may provide further insights into ICI use in the adjuvant setting for high‐risk EC.^15^, ^16^ Additionally, while guidelines recommend combining ICIs with chemotherapy in adjuvant settings for high‐risk endometrial cancer,^4^ optimal sequencing of ICI use requires further exploration in relation to long‐term disease outcomes.

Although clinical trials are gold standards for proving treatment efficacy and safety, their follow‐up periods are typically limited, leaving gaps in understanding long‐term treatment benefits and the associated patient burden. Additionally, trial populations may not be representative in terms of comorbidity profiles, patient fitness, and age. Yet, to date, only a limited number of studies have investigated clinical outcomes, such as DFS and OS, in women with high‐risk EC in real‐world settings.^9^, ^10^, ^11^, ^17^, ^18^ In particular, little is known about disease progression by key prognostic markers, including MMR. To bridge this evidence gap, the objectives of this study were to describe demographic and clinical characteristics and estimate real‐world clinical outcomes of patients with high‐risk EC or endometrial carcinosarcoma from initiation of first adjuvant therapy in England.

## METHODS

This was a retrospective cohort study of adult women with high‐risk EC or endometrial carcinosarcoma in England based on secondary patient‐level data from the National Cancer Registration and Analysis Service (NCRAS) between 2014 and 2023. NCRAS encompasses the Cancer Analysis System Molecular (CAS‐M) for details on biomarker testing of somatic mutations; the Cancer Outcomes and Services Dataset (COSD) for diagnostic and pathological data; the Systemic Anti‐Cancer Therapy (SACT) dataset for NHS‐funded systemic treatment data across hospital inpatient, outpatient, and community settings; the National Radiotherapy Dataset (RTDS) for radiation therapy treatment information; Hospital Episode Statistics (HES) for inpatient admissions, outpatient appointments, and Accident & Emergency attendances; and the Office of National Statistics (ONS) for death statistics. Anonymized study data was prepared by NHS England, and ethics approval was not required.

### Study population

Adult women (≥18 years) with an incident diagnosis of EC or endometrial carcinosarcoma at high risk of recurrence as defined by FIGO criteria^19^ (i.e., FIGO stage I or II with myometrial invasion of non‐endometrioid histology or myometrial invasion of any histology with known aberrant p53 expression or p53 mutation or FIGO stage III or IVA disease)—emulating the patient population in the KEYNOTE‐B21 clinical trial^14^—were identified from the database. Patients were required to have undergone surgery for EC (e.g., hysterectomy and bilateral salpingo‐oophorectomy) and subsequent adjuvant therapy (i.e., radiation, chemo, chemo‐radiation, or hormone therapy) within 90 days after surgery. The date of initiation of first adjuvant therapy was defined as the index date. Patients were excluded if they had missing records of NHS numbers, date of birth, or vital status; received drugs via the Cancer Drugs Fund (CDF); any record of systemic anti‐cancer therapy up to 30 days before the date of EC diagnosis; concomitant malignant primary or secondary neoplasms (excluding non‐melanoma skin cancer) before the date of diagnosis or primary malignant neoplasms post‐index; diagnosis of any uterine cancer other than EC or endometrial carcinosarcoma; and/or record of a POLE abnormality at any time 60 days within the date of diagnosis until end of follow‐up.

### Outcomes

Disease progression and recurrence are not documented within NCRAS. Real‐world disease‐free survival (DFS) was therefore operationalized as time to next treatment or death (TTNTD), measured as time in years from the initiation of first adjuvant therapy until the start date of the next treatment line (i.e., receipt of chemotherapy >90 days or radiotherapy >45 days after end of adjuvant therapy) or surgery, or date of death from any cause. Disease recurrence was defined as (1) a gap >90 days between the adjuvant therapy after surgery for EC (i.e., radiation, chemotherapy, chemo‐radiation, or hormone therapy) and the next chemo, chemo‐radiation, or hormone therapy, or a gap >45 days if the next treatment was radiation therapy, or (2) a record of secondary malignancy after the adjuvant therapy after surgery for EC. OS was measured as time in years from the initiation of first adjuvant therapy until the date of all‐cause death. Patients were censored at the earliest date of loss to follow‐up or the end of the study period.

### Statistical analysis

Descriptive statistics were used to summarize the baseline demographic and clinical characteristics of the cohort. OS and rwDFS were estimated using the Kaplan–Meier estimator for the total cohort and stratified by MMR status (see Supporting Information Section 1 for assessment details), histology, FIGO stage, and Eastern Clinical Oncology Group (ECOG) status at diagnosis. OS was also stratified by recurrence status. A Cox regression model was fitted to evaluate factors, including disease recurrence, associated with OS. The Aalen‐Johansen estimator predicted transition probabilities using a 3‐state model (adjuvant treatment initiation, recurrence, death). A sensitivity analysis was conducted among patients receiving carboplatin + paclitaxel with optional radiotherapy as adjuvant therapy.

The data supporting the findings of this study were prepared by NHS England and are not publicly available due to ethical restrictions (see the Data availability statement for details). In accordance with the journal's guidelines, we will provide our data for independent analysis by a selected team by the Editorial Team for the purposes of additional data analysis or for the reproducibility of this study in other centers if such is requested.

## RESULTS

A total of 77 201 adult women were diagnosed with EC or endometrial carcinosarcoma in NCRAS between January 1, 2012, and December 31, 2021. Of these, 6036 (8%) met the eligibility criteria for inclusion. Mean follow‐up was 48 (SD: 33) months. Demographic and clinical characteristics of the overall cohort are in Table 1. The mean patient age was 67 years (SD: 10), and most (86%) were of White ethnicity. In total, 461 (8%) patients had a MMR biomarker test record (a mean of 106 days after diagnosis). Of these, 115 patients were dMMR (25%) and the remaining 346 (75%) patients were pMMR, with a mean follow‐up of 33 (SD: 9) and 31 (SD: 11) months, respectively. Demographic and clinical characteristics stratified by MMR status, histology, FIGO stage, and ECOG status are provided in the Supporting Information (Tables S1–S4).

**TABLE 1:** Demographic and clinical characteristics of the patient sample.

	Total sample (*n* = 6036)
Age at index^a^, mean (SD) (P_5_–P_95_) years	67.46 (9.98) (50.00–83.00)
Ethnicity	
White	5163 (85.54%)
Black	261 (4.32%)
Asian	263 (4.36%)
Mixed	44 (0.73%)
Chinese/other	118 (1.95%)
Missing	187 (3.10%)
Geographic area	
East Midlands	563 (9.33%)
East of England	800 (13.25%)
London	942 (15.61%)
North East	301 (4.99%)
North West	753 (12.48%)
South East	925 (15.32%)
South West	689 (11.41%)
West Midlands	689 (11.41%)
Yorkshire and The Humber	374 (6.20%)
Missing	0 (0.00%)
Year of diagnosis	
2012	285 (4.72%)
2013	383 (6.35%)
2014	622 (10.30%)
2015	643 (10.65%)
2016	661 (10.95%)
2017	644 (10.67%)
2018	683 (11.32%)
2019	709 (11.75%)
2020	642 (10.64%)
2021	764 (12.66%)
Histology at diagnosis	
Endometrioid carcinoma	2214 (36.68%)
Mucinous carcinoma	57 (0.94%)
Clear cell carcinoma	502 (8.32%)
Serous carcinoma	2326 (38.54%)
Mixed carcinoma	27 (0.45%)
Carcinosarcoma	88 (1.46%)
Missing/unknown	822 (13.62%)
FIGO surgical stage at diagnosis	
I	1553 (25.73%)
II	437 (7.24%)
III	3961 (65.62%)
IVA	85 (1.14%)
ECOG performance status (at diagnosis)	
0	2204 (36.51%)
1	836 (13.85%)
2+	178 (2.95%)
Unknown	2818 (46.69%)
MMR status^b^	
dMMR	115 (1.91%)
pMMR	346 (5.73%)
Missing/unknown^c^	5575 (92.36%)
Disease recurrence during follow‐up	2748 (45.52%)
Site of recurrence due to secondary malignancy	
Locoregional	454 (16.52%)
Distant	1363 (49.60%)
Unknown^d^	931 (33.88%)
Metastasis during follow‐up	2025 (33.55%)
Time from diagnosis to the index date^a^, mean (SD) (P_5_–P_95_) months	3.32 (1.40) (1.77–5.16)
Duration of follow‐up, mean (SD) (P_5_–P_95_) months	48.30 (32.99) (7.19–110.78)
Comorbidities	
CCI score, mean (SD) (P_5_–P_95_) months	0.32 (0.80) (0.00–2.00)
Obesity	1642 (27.20%)
Chronic pulmonary disease	758 (12.56%)
Renal disease	365 (6.05%)

*Note*: Data presented as n (proportion %) unless specified otherwise.

Abbreviations: ECOG, Eastern Clinical Oncology Group; FIGO, International Federation of Gynecology and Obstetrics; MMR, Mismatch Repair.

^a^ Date of initiation of first adjuvant therapy (i.e., radiation, chemo, chemo‐radiation, or hormone therapy) within 90 days of the date of surgery for EC.

^b^ MMR biomarker data capture spans 01/01/2019–31/12/2021.

^c^ Includes patients without CAS Molecular Diagnostics (CAS‐MDx) linkage, those not tested, and patients with insufficient test results.

^d^ Includes patients identified as recurred due to having a gap >90 days between the adjuvant therapy after surgery for EC (i.e., radiation, chemo, chemo‐radiation, or hormone therapy) and the next treatment (i.e., chemo, chemo‐radiation, or hormone therapy), or due to the diagnosis of a secondary malignancy being unspecified.

### Real‐world disease‐free survival (rwDFS)

**FIGURE 1:**
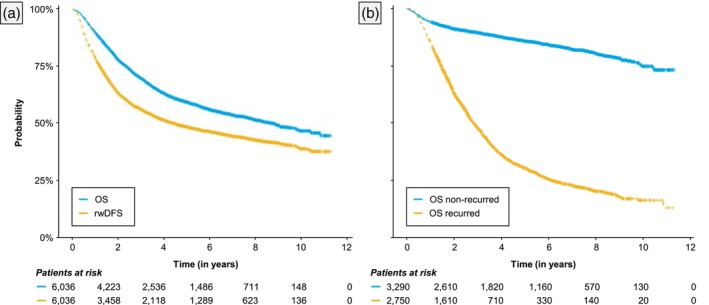
(a) Overall survival and real‐world disease‐free survival from initiation of first adjuvant therapy and (b) Overall survival by recurrence status. Real‐world disease‐free survival was operationalized as time to next treatment or death (TTNTD).

**FIGURE 2:**
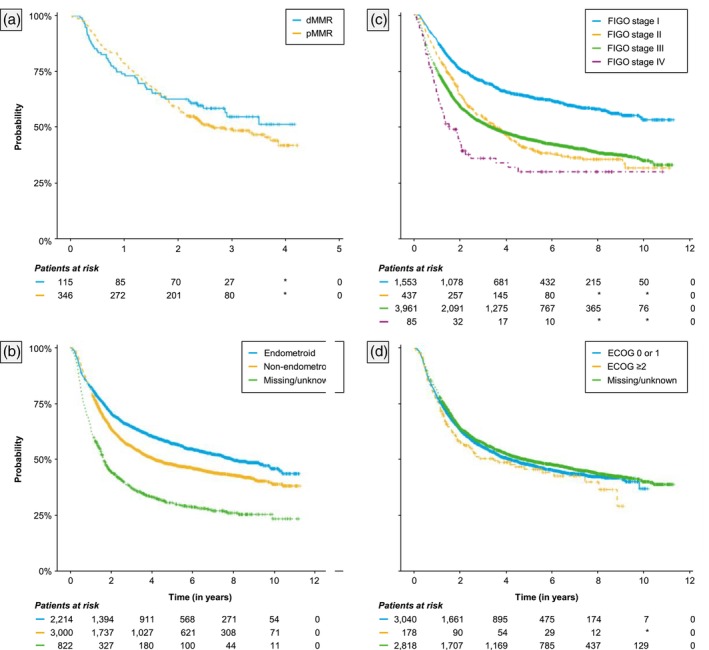
Real‐world disease‐free survival from initiation of first adjuvant therapy, by (a) MMR status, (b) Histology at diagnosis, (c) FIGO stage, and (d) ECOG status. Real‐world disease‐free survival was operationalized as time to next treatment or death (TTNTD).

Estimated Kaplan–Meier survival functions of rwDFS (i.e., TTNTD) from the index date (i.e., initiation of first adjuvant therapy after diagnosis) are presented in Figures 1a and 2. Overall, 45% of patients experienced recurrence during the study period. Median rwDFS from the index date was estimated at 4.56 years (95% CI: 4.14–5.12). Estimated 2‐year and 5‐year rwDFS probabilities were 0.64 (0.63–0.65) and 0.49 (0.48–0.50), respectively. Median rwDFS was not reached (2.48–NA) for dMMR patients, 2.67 years (2.21–NA) for pMMR patients, 4.16 years (3.70–4.96) for patients with non‐endometrioid histology, 7.97 years (6.95–9.80) for patients with endometrioid histology, and 4.26 years (3.59–4.97) among those who received carboplatin + paclitaxel with or without radiotherapy as adjuvant therapy. Additional estimates of rwDFS for examined subgroups are provided in the Supporting Information (Table S5).

### Overall survival (OS)

**FIGURE 3:**
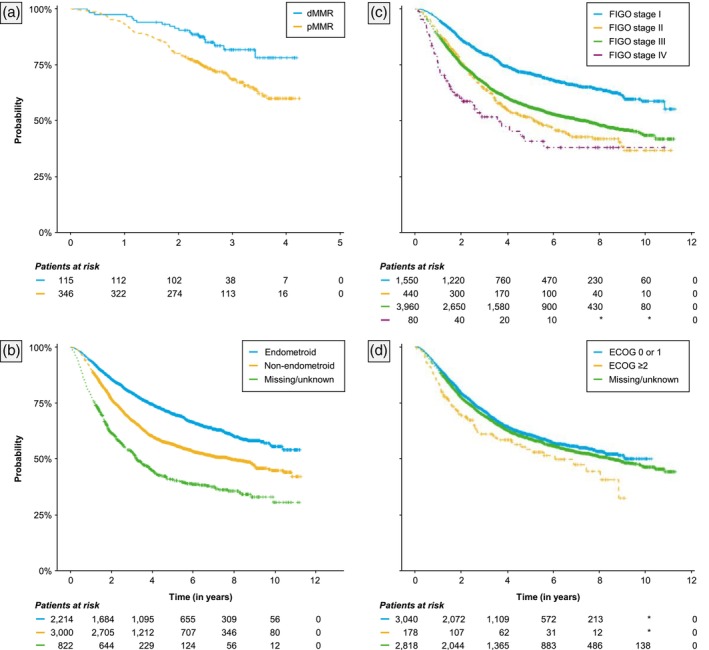
Overall survival from initiation of first adjuvant therapy, by (a) MMR status, (b) Histology at diagnosis, (c) FIGO stage, and (d) ECOG status.

Estimated Kaplan–Meier survival functions of OS from the index date are presented in Figures 1a and 3. Overall, 39% of patients died due to any cause during the study period. Median OS from the index date was estimated at 8.85 years (95% CI: 8.15–9.82). Estimated 2‐year and 5‐year OS survival probabilities were 0.78 (0.77–0.79) and 0.60 (0.58–0.61), respectively. Among those who experienced recurrence, estimated 2‐year and 5‐year OS survival probabilities were 0.63 (0.61–0.65) and 0.30 (0.29–0.32), respectively, and among non‐recurred patients these were 0.91 (0.90–0.92) and 0.86 (0.84–0.87), respectively (Figure 1b). Median OS was not reached among dMMR and pMMR patients, 7.95 years (6.69–9.04) among patients with non‐endometrioid histology, not reached (0.39–NA) among patients with endometrioid histology, and not reached (8.96–NA) among those who received carboplatin + paclitaxel with or without radiotherapy as adjuvant therapy. The Supporting Information contains additional estimates of OS for these subgroups (Table S5), Kaplan–Meier survival functions for patients who received carboplatin + paclitaxel with or without radiotherapy as adjuvant therapy (Figures S1 and S2 [dMMR]), as well as Aalen‐Johnsen estimates of transition probabilities of first disease recurrence and death, respectively (Table S6).

Results from the Cox proportional hazards model of the impact of disease recurrence on OS from the index date are presented in Table 2. Patients who experienced recurrence had a 3.23‐fold higher risk of mortality compared to those who did not experience recurrence.

**TABLE 2:** Cox proportional hazards model of the impact of disease recurrence on overall survival.

	HR	95% CI	*p*‐value
Recurrence status			
Non‐recurred	Reference	–	–
Recurred	3.23	3.02–3.44	<0.001
Age			
<54	Reference	–	–
55–64	1.73	1.49–2	<0.001
65–74	2.31	2.01–2.66	<0.001
75–84	3.05	2.65–3.52	<0.001
85+	3.99	3.22–4.94	<0.001
MMR status			
dMMR	Reference	–	–
Missing/unknown	1.9	1.37–2.64	<0.001
pMMR	1.69	1.18–2.41	0.004
Histology			
Non‐endometrioid	Reference	–	–
Endometrioid	0.51	0.47–0.56	<0.001
Missing/unknown	1	0.91–1.09	0.962
FIGO stage			
III	Reference	–	–
I	0.48	0.43–0.53	<0.001
II	0.83	0.73–0.93	0.002
IV	1.39	1.11–1.73	0.004
Ethnicity			
White	Reference	–	–
Black	1.15	1–1.32	0.055
Asian	0.91	0.77–1.07	0.236
Mixed	1.14	0.82–1.59	0.429
Chinese/other	1.09	0.87–1.38	0.443
Missing/unknown	0.63	0.5–0.8	<0.001
Time from diagnosis to index date (months)	1.03	1.01–1.05	0.011

*Note*: Recurrence status was modeled as a time‐dependent covariate.

### Association between real‐world disease‐free survival and overall survival

Kendall's *τ* correlation coefficient between rwDFS (i.e., TTNTD) and OS was estimated at 0.75 (95% CI: 0.69–0.80, *p* < 0.001).

## DISCUSSION

To our knowledge, this retrospective cohort study is the first to estimate real‐world clinical outcomes in women with high‐risk EC in England. In the total cohort, median rwDFS and OS from initiation of adjuvant therapy were estimated at 4.56 and 8.85 years, respectively. However, outcomes varied significantly across subgroups, including MMR status, histology, and FIGO stage. As expected, disease recurrence was associated with markedly lower OS. Specifically, we found that patients who recurred had 3 times the risk of death compared to those who did not recur, and the 5‐year OS probability was less than half of those who did not recur (0.30 vs. 0.86). Outcomes were also poor for patients who received carboplatin + paclitaxel with or without radiotherapy as adjuvant therapy. Finally, in our cohort, there was a strong and significant correlation between real‐world DFS and OS.

Only a handful of studies have examined real‐world clinical outcomes among women with high‐risk EC. Although not directly comparable due to differences in patient selection and study design, the 3‐year rwDFS estimate of 0.57 in our study was similar to the 3‐year DFS estimates (0.52–0.75) reported in Bendifallah et al.,^10^ a case–control study involving 396 French women. The proportion of patients who experienced recurrence after adjuvant therapy was markedly higher in our study compared to the estimate reported by Prabhu et al.^11^ in their analysis of 1199 women with high‐risk EC receiving adjuvant therapy in the United States (45% vs. 32%). Additionally, compared with Prabhu et al.,^11^ a larger proportion of women in our study had more advanced disease, that is, stage III (66% vs. 22%) compared to stage I (26% vs. 68%). Interestingly, despite higher recurrence rates, median OS for those who recurred in our study was longer than estimates reported in Prabhu et al.^11^ However, for this comparison, it is important to note that the cohort reported in Prabhu et al. comprised older patients due to the age inclusion criterion of ≥66 years at initial diagnosis.^11^

To contextualize our results, it may be helpful to compare our real‐world estimates with those reported from clinical trials investigating a similar high‐risk EC population, namely PORTEC‐3 (comparing adjuvant chemotherapy and radiotherapy vs. pelvic radiotherapy alone),^12^ GOG 258 (comparing chemoradiotherapy vs. chemotherapy alone),^13^ and Keynote‐B21 (comparing adjuvant pembrolizumab plus chemotherapy vs. chemotherapy alone).^14^ Specifically, 5‐year rwDFS was lower in our study compared to the 5‐year failure‐free survival rate in the PORTEC‐3 trial^12^ (49% vs. 69%–77%) and 5‐year relapse‐free survival rate in the GOG 258 trial^13^ (49% vs. 59%). Five‐year OS was also lower in our study compared to the PORTEC‐3 trial^12^ (60% vs. 76%–81%).

Despite limited biomarker record availability from the CAS‐M dataset in our study, dMMR testing results, where available, aligned with previous research indicating that 25%–31% of EC are dMMR.^20^, ^21^ For the dMMR subgroup treated with carboplatin + paclitaxel with optional radiation, 2‐year rwDFS rates were lower than the interim 2‐year DFS reported for the chemotherapy‐only group in the KEYNOTE‐B21 trial (62% vs. 80%). This is likely due to differences in real‐world versus trial populations (concerning, e.g., age and ECOG status). Yet, due to the small sample size of this analysis in our study and the shorter follow‐up of those tested, results should be interpreted with some caution.

In our analysis, we found a strong correlation between rwDFS and OS at 0.75 (*p* < 0.001), providing further support that DFS is a patient‐relevant outcome and a suitable surrogate endpoint for OS in clinical trials in EC, consistent with results from other studies^22^, ^23^ This finding would be expected to be particularly relevant to early settings, where it takes relatively longer to accrue mature OS data.

We also evaluated factors associated with OS (Figure 2 and Table 2). Overall, our findings were consistent with the literature that increased age,^24^ pMMR,^25^ non‐endometrioid histology,^26^ and increased stage^27^ are associated with increased mortality and poorer outcomes. Furthermore, the increased hazard for mortality was three times higher for those aged >75. We also found a trend for women of Black compared with White ethnicity to have significantly shorter OS (HR: 1.15, *p* = 0.055). This result agrees with previous research showing that Black women have comparatively poor cancer outcomes.^28^, ^29^, ^30^ Several reasons have been discussed in the literature, such as barriers in access to diagnosis leading to advanced stage at diagnosis, or barriers to treatment due to cultural or religious beliefs, or being predisposed to histological and molecular EC subtypes that are more aggressive.^30^ Yet, further study of this topic is warranted to help delineate causes and improve the situation.

Key strengths of our study include its generalizability and size, capturing approximately 98% of all cancer patients in England, with high geographic representativeness of individuals receiving public healthcare. However, NCRAS does not record disease recurrence, progression, or treatment response. As a result, rwDFS in our study was proxied using TTNTD, which may systematically underestimate rwDFS (due to, e.g., treatment toxicity, patient characteristics or preference, and/or treatment availability) and/or misclassify delay in treatment with recurrence. Due to data unavailability, we were also unable to adjust our regression analysis for cigarette smoking, and similar to all observational studies, we are unable to infer causality for the identified associations. Finally, CAS‐M biomarker data coverage is known to have an uneven representation for England due to under‐reporting of molecular results in certain areas of England. Therefore, the data may not be geographically representative of all of England. The fact that CAS‐M biomarker data was only available for a subset of the total cohort (8%) also limited the precision of our estimates and possibilities to perform further subgroup analysis. Expanded testing is expected alongside increased awareness of the sensitivity of dMMR tumors to ICIs.^14^, ^31^

The results from our study underscore the substantial clinical burden and unmet medical need of women with high‐risk EC, and the devastating impact of disease recurrence in particular. The variability in real‐world outcomes across clinical subgroups will be helpful to inform treatment strategies and sampling of patients to clinical trials, and patient‐centric prognosis evaluation. Given the increased mortality risk for recurrent patients, it will be important to assess whether ICI usage should be prioritized to prevent recurrence and improve long‐term OS in future treatment sequencing models. Finally, the strong and statistically significant association between rwDFS and OS provides further evidence of the suitability of using the former as a surrogate for the latter.

In conclusion, this study provides novel insights into the real‐world clinical outcomes of patients with high‐risk EC and adds to the growing evidence of the burden of recurrence and the validity of rwDFS as a surrogate for OS.

## AUTHOR CONTRIBUTIONS

**Vimalanand S. Prabhu:** Conceptualization; methodology; writing – review and editing. **Erik Landfeldt:** Conceptualization; methodology; visualization; writing – review and editing; writing – original draft. **Eleanor Ralphs:** Conceptualization; methodology; writing – original draft; writing – review and editing. **Cheryl Teoh:** Conceptualization; methodology; writing – original draft; writing – review and editing. **Jess Ridsdale‐Smith:** Conceptualization; methodology; writing – original draft; writing – review and editing. **Karen Macey:** Methodology; conceptualization; writing – review and editing. **Nikolay Trankov:** Methodology; formal analysis; writing – review and editing; visualization; data curation. **Alexandrina Lambova:** Methodology; formal analysis; visualization; writing – review and editing; data curation. **Jasmine Lichfield:** Conceptualization; methodology; writing – review and editing. **Gemma Eminowicz:** Conceptualization; methodology; writing – review and editing.

## CONFLICT OF INTEREST STATEMENT

Eleanor Ralphs, Jess Ridsdale‐Smith, Cheryl Teoh, Erik Landfeldt, Nikolay Trankov, and Alexandrina Lambova are employees of IQVIA, a contract research organization. Vimalanand S. Prabhu, Jasmine Lichfield, and Karen Macey are employees of Merck Sharp & Dohme LLC, a subsidiary of Merck & Co., Inc., Rahway, NJ, USA. Gemma Eminowicz reports consulting fees from MSD and Eisai; payment or honoraria from Eisai and GSK; support for attending meetings from MSD; and participation on the Data Safety Monitoring Board or Advisory Board for GSK, MSD, and Eisai, outside the submitted work.

## ETHICS STATEMENT

The study data were collected by NHS England and anonymized; so ethics approval was not required.

## Supporting information


**Table S1.** Demographic and clinical characteristics of the patient sample by MMR status.
**Table S2.** Demographic and clinical characteristics of the patient sample by histology.
**Table S3.** Demographic and clinical characteristics of the patient sample by FIGO stage.
**Table S4.** Demographic and clinical characteristics of the patient sample by ECOG status.
**Table S5.** Real‐world disease‐free survival and overall survival stratified by MMR status, histology, FIGO stage, ECOG, recurrence status, and carboplatin + paclitaxel +/−RT adjuvant therapy.
**Table S6.** Aalen‐Johnsen estimates of probabilities of first disease recurrence and death, respectively, from date of initiation of adjuvant therapy, as well as probabilities of death from disease recurrence.
**Figure S1.** (A) Real‐world disease‐free survival and (B) Overall survival from initiation of first adjuvant therapy, restricted to patients receiving carboplatin + paclitaxel +/−RT as adjuvant therapy.
**Figure S2.** (A) Real‐world disease‐free survival and (B) Overall survival from initiation of first adjuvant therapy, restricted to patients receiving carboplatin + paclitaxel +/−RT as adjuvant therapy and dMMR.

## Data Availability

This work uses data that have been provided by patients and collected by the National Health Service (NHS) England as part of their care and support. The data are collated, maintained, and quality assured by the National Disease Registration Service (NDRS), which is part of NHS England. Access to this data was facilitated by the Simulacrum produced by Health Data Insight CIC. As part of NHS England, the NDRS has a special legal instruction to collect patient data without needing informed consent. This instruction is granted under section 254 of the Health and Social Care Act 2012. NCRAS data are not publicly available, and access must meet strict governance standards. Formal requests for the release of NCRAS data are managed by NHS England's Data Access Request Service (DARS) (available at: https://digital.nhs.uk/services/data-access-request-service-dars).
