# Supplementary material for: Real‐world clinical outcomes of patients with high‐risk endometrial cancer or endometrial carcinosarcoma in England: A retrospective cohort study
Source: J Obstet Gynaecol Res. 2025 Sep 19;51(9):e70042. doi: 10.1111/jog.70042 (PMC12447258; doi:10.1111/jog.70042)
Supplement: Supplementary file 1 — Table S1. Demographic and clinical characteristics of the patient sample by MMR status. Table S2. Demographic and clinical characteristics of the patient sample by histology. Table S3. Demographic and clinical characteristics of the patient sample by FIGO stage. Table S4. Demographic and clinical characteristics of the patient sample by ECOG status. Table S5. Real‐world disease‐free survival and overall survival stratified by MMR status, histology, FIGO stage, ECOG, recurrence status, and carboplatin + paclitaxel +/−RT adjuvant therapy. Table S6. Aalen‐Johnsen estimates of probabilities of first disease recurrence and death, respectively, from date of initiation of adjuvant therapy, as well as probabilities of death from disease recurrence. Figure S1. (A) Real‐world disease‐free survival and (B) Overall survival from initiation of first adjuvant therapy, restricted to patients receiving carboplatin + paclitaxel +/−RT as adjuvant therapy. Figure S2. (A) Real‐world disease‐free survival and (B) Overall survival from initiation of first adjuvant therapy, restricted to patients receiving carboplatin + paclitaxel +/−RT as adjuvant therapy and dMMR. [file JOG-51-0-s001.docx]

# Supplementary Materials

*Section 1: Assessment of MMR status*

Patients were categorized as either dMMR (deficiency of MMR function); pMMR (proficient MMR); or unknown where not enough data on biomarker test results were available for a given patient. If a patient had an ‘abnormal’ result recorded under OVERALL_TS for ANY of the following four genes: MLH1, MSH2, MSH6, PMS2; then they were categorized as dMMR. If a patient had a ‘normal’ test result recorded for ANY of the four genes above, AND they had no ‘abnormal’ test result recorded for any of these four genes, then they were categorized as pMMR. All other patients were categorized as ‘unknown MMR status’.

**Table S1: Demographic and clinical characteristics of the patient sample by MMR status^a^**

|  | **pMMR**  **(n=346)** | **dMMR**  **(n=115)** |
| --- | --- | --- |
| **Age at index^b^, mean (SD) (P_5_­–P_95_) years** | 65.8 (10.84) (48.00 - 81.00) | 63.30 (10.51) (47.00 - 79.30) |
| **Ethnicity** | |  |
| White | 281 (81.21%) | 94 (81.74%) |
| Black | ** (**) | * (*%) |
| Asian | 18 (5.20%) | 7 (6.09%) |
| Mixed | * (*%) | ** (**) |
| Chinese/Other | ** (**) | * (*%) |
| Missing | 6 (1.73%) | 6 (5.22%) |
| **Geographic area** | |  |
| East Midlands | 31 (8.96%) | 7 (6.09%) |
| East of England | 57 (16.47%) | 10 (8.70%) |
| London | 84 (24.28%) | 29 (25.22%) |
| North East | ** (**) | ** (**) |
| North West | 16 (4.62%) | 12 (10.43%) |
| South East | 78 (22.54%) | 22 (19.13%) |
| South West | 35 (10.12%) | 14 (12.17%) |
| West Midlands | 23 (6.65%) | 13 (11.30%) |
| Yorkshire and The Humber | ** (**) | * (*%) |
| Missing | 0 (0.00%) | 0 (0.00%) |
| **Year of diagnosis** | |  |
| 2019 | 136 (39.31%) | 41 (35.65%) |
| 2020 | 210 (60.69%) | 74 (64.35%) |
| 2021 | 0 (0.00%) | 0 (0.00%) |
| **Histology at diagnosis** | |  |
| Endometroid | 116 (33.53%) | 86 (74.78%) |
| Non-endometroid | 182 (52.60%) | 11 (9.57%) |
| Missing/Unknown | 48 (13.87%) | 18 (15.65%) |
| **FIGO surgical stage at diagnosis** | |  |
| I | 69 (19.94%) | 7 (6.09%) |
| II | 24 (6.94%) | 0 (0.00%) |
| III | 233 (67.34%) | 103 (89.57%) |
| IVA | * (*%) | * (*%) |
| **ECOG performance status (at diagnosis)** | |  |
| 0-1 | 205 (59.25%) | 60 (52.17%) |
| 2+ | 7 (2.02%) | * (*%) |
| Unknown | 134 (38.73%) | 51 (44.35%) |
| **Disease recurrence during follow-up** | 185 (53.47%) | 55 (47.83%) |
| **Site of disease recurrence** | |  |
| Locoregional recurrence | 27 (7.80%) | 11 (9.57%) |
| Distant recurrence | 94 (27.17%) | 18 (15.65%) |
| Recurrence of unknown site | 64 (18.50%) | 26 (22.61%) |
| No record of recurrence | 161 (46.53%) | 60 (52.17%) |
| **Metastasis during follow-up** | 2,025 (33.55%) |  |
| **Time from diagnosis to initiation of first adjuvant therapy (i.e., the index date^c^), mean (SD) (P_5_­–P_95_) months** | 3.43 (1.64) (1.85 - 5.65) | 3.72 (1.83) (1.76 - 6.25) |
| **Duration of follow-up, mean (SD) (P_5_­–P_95_) months** | 30.95 (10.89) (10.23 - 47.79) | 32.75 (9.46) (14.45 - 49.24) |
| **Comorbidities^c^** | |  |
| CCI score, mean (SD) (P_5_­–P_95_) months | 0.28 (0.74) (0.00 - 2.00) | 0.23 (0.58) (0.00 - 1.00) |
| Obesity | 118 (34.10%) | 32 (27.83%) |
| Chronic pulmonary disease | 43 (12.43%) | 16 (13.91%) |
| Renal disease | 15 (4.34%) | 6 (5.22%) |

*Note: Data presented as n (proportion %) unless specified otherwise.*

*Abbreviations: ECOG = Eastern Clinical Oncology Group, FIGO = International Federation of Gynecology and Obstetrics, MMR = Mismatch Repair.*

*^a^ MMR biomarker data capture spans 01/01/2019 - 31/12/2021. ^b^ Date of initiation of first adjuvant therapy (i.e., radiation, chemo, chemo-radiation, or hormone therapy) within 90 days of the date of surgery for EC. ^c^ Figures reported should be considered as minimum estimates due to the possibility of missing or incomplete records. * All small numbers 1-5. ** Secondary data suppression was applied as needed to prevent backwards derivation of small numbers.*

**Table S2: Demographic and clinical characteristics of the patient sample by histology**

|  | **Endometrioid**  **(n=2,214)** | **Non-endometroid**  **(n=3,000)** | | **Missing**  **(n=822)** |
| --- | --- | --- | --- | --- |
| **Age at index^a^, mean (SD) (P_5_­–P_95_) years** | 64.15 (10.93) (47.00 - 82.00) | 69.89 (8.29) (56.00 - 83.00) | | 67.55 (10.36) (50.05 - 82.00) |
| **Ethnicity** |  | |  | |
| White | 1954 (88.26%) | 2519 (83.97%) | | 690 (83.94%) |
| Black | 34 (1.54%) | 186 (6.20%) | | 41 (4.99%) |
| Asian | 81 (3.66%) | 145 (4.83%) | | 37 (4.50%) |
| Mixed | 11 (0.50%) | 24 (0.80%) | | 9 (1.09%) |
| Chinese/Other | 50 (2.26%) | 49 (1.63%) | | 19 (2.31%) |
| Missing | 84 (3.79%) | 77 (2.57%) | | 26 (3.16%) |
| **Geographic area** |  | |  | |
| East Midlands | 191 (8.63%) | 292 (9.73%) | | 80 (9.73%) |
| East of England | 277 (12.51%) | 435 (14.50%) | | 88 (10.71%) |
| London | 291 (13.14%) | 511 (17.03%) | | 140 (17.03%) |
| North East | 107 (4.83%) | 156 (5.20%) | | 38 (4.62%) |
| North West | 329 (14.86%) | 312 (10.40%) | | 112 (13.63%) |
| South East | 356 (16.08%) | 437 (14.57%) | | 132 (16.06%) |
| South West | 264 (11.92%) | 350 (11.67%) | | 75 (9.12%) |
| West Midlands | 255 (11.52%) | 332 (11.07%) | | 102 (12.41%) |
| Yorkshire and The Humber | 144 (6.50%) | 175 (5.83%) | | 55 (6.69%) |
| Missing | 0 (0.00%) | 0 (0.00%) | | 0 (0.00%) |
| **Year of diagnosis** |  | |  | |
| 2012 | 102 (4.61%) | 144 (4.80%) | | 39 (4.74%) |
| 2013 | 161 (7.27%) | 186 (6.20%) | | 36 (4.38%) |
| 2014 | 238 (10.75%) | 298 (9.93%) | | 86 (10.46%) |
| 2015 | 249 (11.25%) | 317 (10.57%) | | 77 (9.37%) |
| 2016 | 228 (10.30%) | 347 (11.57%) | | 86 (10.46%) |
| 2017 | 236 (10.66%) | 323 (10.77%) | | 85 (10.34%) |
| 2018 | 222 (10.03%) | 359 (11.97%) | | 102 (12.41%) |
| 2019 | 235 (10.61%) | 368 (12.27%) | | 106 (12.90%) |
| 2020 | 249 (11.25%) | 298 (9.93%) | | 95 (11.56%) |
| 2021 | 294 (13.28%) | 360 (12.00%) | | 110 (13.38%) |
| **FIGO surgical stage at diagnosis** |  | |  | |
| I | 0 (0.00%) | 487 (16.23%) | | * (*%) |
| II | 0 (0.00%) | ** (**) | | * (*%) |
| III | 2181 (98.51%) | 985 (32.83%) | | 795 (96.72%) |
| IVA | 33 (1.49%) | 28 (0.93%) | | 24 (2.92%) |
| **ECOG performance status (at diagnosis)** |  | |  | |
| 0-1 | 1130 (51.03%) | 1480 (49.34%) | | 430 (52.31%) |
| 2+ | 68 (3.07%) | 87 (2.90%) | | 23 (2.80%) |
| Unknown | 1016 (45.89%) | 1433 (47.77%) | | 369 (44.89%) |
| **MMR status^b^** |  | |  | |
| dMMR | 86 (3.88%) | 11 (0.37%) | | 18 (2.19%) |
| pMMR | 116 (5.24%) | 182 (6.07%) | | 48 (5.84%) |
| Missing/Unknown^c^ | 2012 (90.88%) | 2807 (93.57%) | | 756 (91.97%) |
| **Disease recurrence during follow-up** | 923 (41.69%) | 1339 (44.63%) | | 486 (59.12%) |
| **Site of disease recurrence** |  | |  | |
| Locoregional recurrence | 186 (8.40%) | 199 (6.63%) | | 69 (8.39%) |
| Distant recurrence | 406 (18.34%) | 698 (23.27%) | | 259 (31.51%) |
| Recurrence of unknown site | 331 (14.95%) | 442 (14.73%) | | 158 (19.22%) |
| No record of recurrence | 1291 (58.31%) | 1661 (55.37%) | | 336 (40.88%) |
| **Metastasis during follow-up** | 2,025 (33.55%) |  | |  |
| **Time from diagnosis to initiation of first adjuvant therapy (i.e., the index date^c^), mean (SD) (P_5_­–P_95_) months** | 3.15 (2.56-3.84) (2214) | 3.19 (2.69-3.71) (3000) | | 3.12 (2.60-3.65) (822) |
| **Duration of follow-up, mean (SD) (P_5_­–P_95_) months** | 53.41 (33.24) (10.05 - 112.54) | 47.59 (32.66) (8.05 - 110.96) | | 37.12 (30.52) (4.05 - 101.29) |
| **Comorbidities^d^** |  | |  | |
| CCI score, mean (SD) (P_5_­–P_95_) months | 0.28 (0.77) (0.00 - 2.00) | 0.33 (0.79) (0.00 - 2.00) | | 0.36 (0.87) (0.00 - 2.00) |
| Obesity | 681 (30.76%) | 749 (24.97%) | | 212 (25.79%) |
| Chronic pulmonary disease | 263 (11.88%) | 365 (12.17%) | | 130 (15.82%) |
| Renal disease | 126 (5.69%) | 186 (6.20%) | | 53 (6.45%) |

*Note: Data presented as n (proportion %) unless specified otherwise.*

*Abbreviations: ECOG = Eastern Clinical Oncology Group, FIGO = International Federation of Gynecology and Obstetrics, MMR = Mismatch Repair.*

*^a^ Date of initiation of first adjuvant therapy (i.e., radiation, chemo, chemo-radiation, or hormone therapy) within 90 days of the date of surgery for EC. ^b^ MMR biomarker data capture spans 01/01/2019 - 31/12/2021. ^c^ Includes a mix of patients, i.e., those without CAS Molecular Diagnostics (CAS-MDx) linkage, those not tested or patients with insufficient test results. ^d^ Figures reported should be considered as minimum estimates due to the possibility of missing or incomplete records. * All small numbers 1-5. ** Secondary data suppression was applied as needed to prevent backwards derivation of small numbers.*

**Table S3: Demographic and clinical characteristics of the patient sample by FIGO stage**

|  | **I**  **(n=1,553)** | **II**  **(n=437)** | | **III**  **(n=3,961)** | | **IV**  **(n=85)** |
| --- | --- | --- | --- | --- | --- | --- |
| **Age at index^a^, mean (SD) (P_5_­–P_95_) years** | 69.60 (8.20) (56.00 - 83.00) | 70.19 (8.73) (55.00 - 83.20) | | 66.37 (10.51) (48.00 - 82.00) | | 65.31 (11.12) (48.80 - 82.00) |
| **Ethnicity** |  | |  | |  | |
| White | 1291 (83.13%) | 369 (84.44%) | | 3430 (86.59%) | | 73 (85.88%) |
| Black | 96 (6.18%) | ** (**) | | 136 (3.43%) | | * (*%) |
| Asian | 83 (5.34%) | ** (**) | | 158 (3.99%) | | * (*%) |
| Mixed | 14 (0.90%) | * (*%) | | 27 (0.68%) | | * (*%) |
| Chinese/Other | 26 (1.67%) | 7 (1.60%) | | 85 (2.15%) | | 0 (0.00%) |
| Missing | 43 (2.77%) | ** (**) | | 125 (3.16%) | | * (*%) |
| **Geographic area** |  | |  | |  | |
| East Midlands | 163 (10.50%) | ** (**) | | 360 (9.09%) | | ** (**) |
| East of England | 245 (15.78%) | 63 (14.42%) | | 482 (12.17%) | | 10 (11.76%) |
| London | 284 (18.29%) | 64 (14.65%) | | 583 (14.72%) | | 11 (12.94%) |
| North East | 90 (5.80%) | 23 (5.26%) | | 181 (4.57%) | | 7 (8.24%) |
| North West | 149 (9.59%) | 53 (12.13%) | | 540 (13.63%) | | 11 (12.94%) |
| South East | 210 (13.52%) | 61 (13.96%) | | 632 (15.96%) | | 22 (25.88%) |
| South West | 171 (11.01%) | 61 (13.96%) | | 450 (11.36%) | | 7 (8.24%) |
| West Midlands | 166 (10.69%) | 48 (10.98%) | | 467 (11.79%) | | 8 (9.41%) |
| Yorkshire and The Humber | 75 (4.83%) | ** (**) | | 266 (6.72%) | | * (*%) |
| Missing | 0 (0.00%) | 0 (0.00%) | | 0 (0.00%) | | 0 (0.00%) |
| **Year of diagnosis** |  | |  | |  | |
| 2012 | 77 (4.96%) | ** (**) | | 179 (4.52%) | | * (*%) |
| 2013 | 98 (6.31%) | ** (**) | | 249 (6.29%) | | * (*%) |
| 2014 | 165 (10.62%) | 37 (8.47%) | | 411 (10.38%) | | 9 (10.59%) |
| 2015 | 156 (10.05%) | 52 (11.90%) | | 427 (10.78%) | | 8 (9.41%) |
| 2016 | 175 (11.27%) | 59 (13.50%) | | 412 (10.40%) | | 15 (17.65%) |
| 2017 | 158 (10.17%) | 49 (11.21%) | | 429 (10.83%) | | 8 (9.41%) |
| 2018 | 177 (11.40%) | 51 (11.67%) | | 448 (11.31%) | | 7 (8.24%) |
| 2019 | 196 (12.62%) | 56 (12.81%) | | 451 (11.39%) | | 6 (7.06%) |
| 2020 | 169 (10.88%) | 33 (7.55%) | | 428 (10.81%) | | 12 (14.12%) |
| 2021 | 182 (11.72%) | 39 (8.92%) | | 527 (13.30%) | | 16 (18.82%) |
| **Histology at diagnosis** |  | |  | |  | |
| Endometroid | 0 (0.00%) | 0 (0.00%) | | 2181 (55.06%) | | 33 (38.82%) |
| Non-endometroid | ** (**) | ** (**) | | 985 (24.87%) | | 28 (32.94%) |
| Missing/Unknown | * (*%) | * (*%) | | 795 (20.07%) | | 24 (28.24%) |
| **ECOG performance status (at diagnosis)** |  | |  | |  | |
| 0-1 | 749 (48.23%) | 151 (34.55%) | | 2029 (51.23%) | | 31 (36.47%) |
| 2+ | 37 (2.38%) | ** (**) | | 122 (3.08%) | | * (*%) |
| Unknown | 767 (49.39%) | 203 (46.45%) | | 1810 (45.70%) | | 38 (44.71%) |
| **MMR status^b^** |  | |  | |  | |
| dMMR | 9 (0.58%) | ** (**) | | 103 (2.60%) | | * (*%) |
| pMMR | 86 (5.54%) | ** (**) | | 233 (5.88%) | | * (*%) |
| Missing/Unknown^c^ | 1458 (93.88%) | 413 (94.51%) | | 3625 (91.52%) | | 79 (92.94%) |
| **Disease recurrence during follow-up** | 469 (30.19%) | 205 (46.91%) | | 2019 (50.98%) | | 55 (64.70%) |
| **Site of disease recurrence** |  | |  | |  | |
| Locoregional recurrence | 67 (4.31%) | 31 (7.09%) | | 350 (8.84%) | | 6 (7.06%) |
| Distant recurrence | 228 (14.68%) | 105 (24.03%) | | 997 (25.17%) | | 33 (38.82%) |
| Recurrence of unknown site | 174 (11.20%) | 69 (15.79%) | | 672 (16.97%) | | 16 (18.82%) |
| No record of recurrence | 1084 (69.80%) | 232 (53.09%) | | 1942 (49.03%) | | 30 (35.29%) |
| **Metastasis during follow-up** | 316 (20.35%) | 143 (32.72%) | | 1513 (38.20%) | | 53 (62.35%) |
| **Time from diagnosis to initiation of first adjuvant therapy (i.e., the index date^c^), mean (SD) (P_5_­–P_95_) months** | 3.30 (1.14) (1.97 - 4.83) | 3.32 (1.02) (1.90 - 5.06) | | 3.32 (1.41) (1.68 - 5.26) | | 4.01 (4.09) (1.36 - 9.54) |
| **Duration of follow-up, mean (SD) (P_5_­–P_95_) months** | 54.68 (33.15) (12.52 - 114.58) | 46.96 (31.62) (8.04 - 107.32) | | 46.22 (32.76) (6.47 - 109.34) | | 35.28 (31.33) (4.42 - 101.36) |
| **Comorbidities^d^** |  | |  | |  | |
| CCI score, mean (SD) (P_5_­–P_95_) months | 0.32 (0.75) (0.00 - 2.00) | 0.41 (0.89) (0.00 - 2.00) | | 0.31 (0.80) (0.00 - 2.00) | | 0.29 (0.77) (0.00 - 2.00) |
| Obesity | 380 (24.47%) | 112 (25.63%) | | 1131 (28.55%) | | 19 (22.35%) |
| Chronic pulmonary disease | 201 (12.94%) | 45 (10.30%) | | 504 (12.72%) | | 8 (9.41%) |
| Renal disease | 92 (5.92%) | 34 (7.78%) | | 233 (5.88%) | | 6 (7.06%) |

*Note: Data presented as n (proportion %) unless specified otherwise.*

*Abbreviations: ECOG = Eastern Clinical Oncology Group, FIGO = International Federation of Gynecology and Obstetrics, MMR = Mismatch Repair.*

*^a^ Date of initiation of first adjuvant therapy (i.e., radiation, chemo, chemo-radiation, or hormone therapy) within 90 days of the date of surgery for EC. ^b^ MMR biomarker data capture spans 01/01/2019 - 31/12/2021. ^c^ Includes a mix of patients, i.e., those without CAS Molecular Diagnostics (CAS-MDx) linkage, those not tested or patients with insufficient test results.* *^d^ Figures reported should be considered as minimum estimates due to the possibility of missing or incomplete records. * All small numbers 1-5. ** Secondary data suppression was applied as needed to prevent backwards derivation of small numbers.*

**Table S4: Demographic and clinical characteristics of the patient sample by ECOG status**

|  | **0-1**  **(n=3,040)** | **2+**  **(n=178)** | | **Unknown**  **(n=2,818)** |
| --- | --- | --- | --- | --- |
| **Age at index^a^, mean (SD) (P_5_­–P_95_) years** | 66.95 (9.76) (50.00 - 81.00) | 72.88 (9.65) (54.00 - 86.00) | | 67.68 (10.12) (50.00 - 83.00) |
| **Ethnicity** |  | |  | |
| White | 2598 (85.46%) | 150 (84.27%) | | 2415 (85.70%) |
| Black | 124 (4.08%) | 7 (3.93%) | | 130 (4.61%) |
| Asian | 132 (4.34%) | ** (**) | | ** (**) |
| Mixed | 22 (0.72%) | 0 (0.00%) | | 22 (0.78%) |
| Chinese/Other | 67 (2.20%) | * (*%) | | ** (**) |
| Missing | 97 (3.19%) | 8 (4.49%) | | 82 (2.91%) |
| **Geographic area** |  | |  | |
| East Midlands | 204 (6.71%) | 12 (6.74%) | | 347 (12.31%) |
| East of England | 445 (14.64%) | 20 (11.24%) | | 335 (11.89%) |
| London | 456 (15.00%) | 23 (12.92%) | | 463 (16.43%) |
| North East | 127 (4.18%) | 10 (5.62%) | | 164 (5.82%) |
| North West | 538 (17.70%) | 35 (19.66%) | | 180 (6.39%) |
| South East | 463 (15.23%) | 35 (19.66%) | | 427 (15.15%) |
| South West | 300 (9.87%) | 10 (5.62%) | | 379 (13.45%) |
| West Midlands | 263 (8.65%) | 18 (10.11%) | | 408 (14.48%) |
| Yorkshire and The Humber | 244 (8.03%) | 15 (8.43%) | | 115 (4.08%) |
| Missing | 0 (0.00%) | 0 (0.00%) | | 0 (0.00%) |
| **Year of diagnosis** |  | |  | |
| 2012 | 0 (0.00%) | 0 (0.00%) | | 285 (10.11%) |
| 2013 | 110 (3.62%) | 7 (3.93%) | | 266 (9.44%) |
| 2014 | 230 (7.57%) | 15 (8.43%) | | 377 (13.38%) |
| 2015 | 291 (9.57%) | 16 (8.99%) | | 336 (11.92%) |
| 2016 | 343 (11.28%) | 22 (12.36%) | | 296 (10.50%) |
| 2017 | 332 (10.92%) | 24 (13.48%) | | 288 (10.22%) |
| 2018 | 359 (11.81%) | 20 (11.24%) | | 304 (10.79%) |
| 2019 | 431 (14.18%) | 22 (12.36%) | | 256 (9.08%) |
| 2020 | 414 (13.62%) | 21 (11.80%) | | 207 (7.35%) |
| 2021 | 530 (17.43%) | 31 (17.42%) | | 203 (7.20%) |
| **Histology at diagnosis** |  | |  | |
| Endometroid | 1130 (37.17%) | 68 (38.20%) | | 1016 (36.05%) |
| Non-endometroid | 1480 (48.68%) | 87 (48.88%) | | 1433 (50.85%) |
| Missing/Unknown | 430 (14.14%) | 23 (12.92%) | | 369 (13.09%) |
| **FIGO surgical stage at diagnosis** |  | |  | |
| I | 525 (17.27%) | 26 (14.61%) | | 766 (27.18%) |
| II | 218 (7.17%) | 16 (8.99%) | | 203 (7.20%) |
| III | 2029 (66.74%) | 122 (68.54%) | | 1810 (64.23%) |
| IVA | 44 (1.45%) | * (*%) | | ** (**) |
| **MMR status^b^** |  | |  | |
| dMMR | 60 (1.97%) | * (*%) | | ** (**) |
| pMMR | 205 (6.74%) | ** (**) | | ** (**) |
| Missing/Unknown^c^ | 2775 (91.28%) | 167 (93.82%) | | 2633 (93.44%) |
| **Disease recurrence during follow-up** | 1424 (46.84%) | 74 (41.57%) | | 1250 (44.36%) |
| **Site of disease recurrence** |  | |  | |
| Locoregional recurrence | 242 (7.96%) | 12 (6.74%) | | 200 (7.10%) |
| Distant recurrence | 684 (22.50%) | 40 (22.47%) | | 639 (22.68%) |
| Recurrence of unknown site | 498 (16.38%) | 22 (12.36%) | | 411 (14.58%) |
| No record of recurrence | 1616 (53.16%) | 104 (58.43%) | | 1568 (55.64%) |
| **Metastasis during follow-up** | 1042 (34.28%) | 54 (30.34%) | | 929 (32.97%) |
| **Time from diagnosis to initiation of first adjuvant therapy (i.e., the index date^c^), mean (SD) (P_5_­–P_95_) months** | 3.40 (1.46) (1.87 - 5.26) | 3.70 (1.26) (1.90 - 5.82) | | 3.22 (1.33) (1.68 - 4.96) |
| **Duration of follow-up, mean (SD) (P_5_­–P_95_) months** | 43.63 (28.77) (7.98 - 100.57) | 39.37 (29.30) (5.08 - 98.02) | | 53.89 (36.41) (7.03 - 119.85) |
| **Comorbidities^d^** |  | |  | |
| CCI score, mean (SD) (P_5_­–P_95_) months | 0.29 (0.73) (0.00 - 2.00) | 0.81 (1.26) (0.00 - 3.00) | | 0.32 (0.81) (0.00 - 2.00) |
| Obesity | 883 (29.05%) | 67 (37.64%) | | 692 (24.56%) |
| Chronic pulmonary disease | 371 (12.20%) | 46 (25.84%) | | 341 (12.10%) |
| Renal disease | 178 (5.86%) | 28 (15.73%) | | 159 (5.64%) |

*Note: Data presented as n (proportion %) unless specified otherwise.*

*Abbreviations: ECOG = Eastern Clinical Oncology Group, FIGO = International Federation of Gynecology and Obstetrics, MMR = Mismatch Repair.*

*^a^ Date of initiation of first adjuvant therapy (i.e., radiation, chemo, chemo-radiation, or hormone therapy) within 90 days of the date of surgery for EC. ^b^ MMR biomarker data capture spans 01/01/2019 - 31/12/2021. ^c^ Includes a mix of patients, i.e., those without CAS Molecular Diagnostics (CAS-MDx) linkage, those not tested or patients with insufficient test results. ^d^ Figures reported should be considered as minimum estimates due to the possibility of missing or incomplete records. * All small numbers 1-5. ** Secondary data suppression was applied as needed to prevent backwards derivation of small numbers.*

**Table S5: Real-world disease-free survival and overall survival stratified by MMR status, histology, FIGO stage, ECOG, recurrence status, and carboplatin + paclitaxel +/-RT adjuvant therapy**

|  | | **Real-world disease-free survival (rwDFS)** | | | **Overall survival (OS)** | | |
| --- | --- | --- | --- | --- | --- | --- | --- |
|  |  | Median (95% CI) | 2-year probability | 5-year probability | Median (95% CI) | 2-year probability | 5-year probability |
| **MMR status** | dMMR (n=115) | Not reached (2.48,NA) | 0.63 (0.54,0.72) | 0.51 (0.41,0.64) | Not reached (NA,NA) | 0.90 (0.85,0.96) | 0.78 (0.69,0.89) |
|  | pMMR (n=346) | 2.67 (2.21,NA) | 0.59 (0.54,0.64) | 0.42 (0.35,0.50) | Not reached (NA,NA) | 0.80 (0.76,0.84) | 0.60 (0.54,0.67) |
| **Histology at diagnosis** | Endometroid (n=2,214) | 7.97 (6.95,9.8) | 0.71 (0.69,0.73) | 0.57 (0.55,0.59) | Not reached (10.39,NA) | 0.86 (0.84,0.87) | 0.70 (0.68,0.72) |
|  | Non-endometroid (n=3,000) | 4.16 (3.7,4.96) | 0.64 (0.62,0.66) | 0.48 (0.46,0.50) | 7.95 (6.69,9.04) | 0.77 (0.75,0.78) | 0.57 (0.55,0.59) |
|  | Missing/Unknown (n=822) | 1.65 (1.51,1.84) | 0.45 (0.41,0.48) | 0.31 (0.28,0.34) | 3.24 (3,3.88) | 0.62 (0.59,0.65) | 0.41 (0.38,0.45) |
| **FIGO stage** | I (n=1,553) | Not reached (9.92,NA) | 0.76 (0.74,0.78) | 0.64 (0.61,0.66) | Not reached (10.83,NA) | 0.87 (0.85,0.88) | 0.71 (0.69,0.74) |
|  | II (n=437) | 3.55 (2.98,4.2) | 0.64 (0.60,0.69) | 0.41 (0.36,0.46) | 5.31 (4.05,6.69) | 0.76 (0.72,0.80) | 0.52 (0.47,0.57) |
|  | III (n=3,961) | 3.34 (3.03,3.72) | 0.59 (0.58,0.61) | 0.44 (0.43,0.46) | 7.53 (6.57,8.27) | 0.75 (0.74,0.77) | 0.56 (0.55,0.58) |
|  | IVA (n=85) | 1.54 (1.15,2.48) | 0.43 (0.34,0.56) | 0.30 (0.21,0.43) | 3.57 (2.02,NA) | 0.60 (0.51,0.72) | 0.41 (0.30,0.56) |
| **ECOG status** | 0-1 (n=3,040) | 4.29 (3.72,5.05) | 0.64 (0.62,0.65) | 0.48 (0.46,0.50) | Not reached (8.25,NA) | 0.79 (0.78,0.81) | 0.61 (0.59,0.63) |
|  | 2+ (n=178) | 3.44 (2.15,8.04) | 0.59 (0.52,0.66) | 0.46 (0.39,0.55) | 6.07 (4.24,NA) | 0.70 (0.63,0.77) | 0.54 (0.46,0.63) |
|  | Unknown (n=2,818) | 4.99 (4.25,6.04) | 0.64 (0.62,0.66) | 0.50 (0.48,0.52) | 8.56 (7.66,9.76) | 0.77 (0.76,0.79) | 0.59 (0.57,0.61) |
| **Recurrence status** | Non-recurred (3,288) | - | - | - | Not reached (NA,NA) | 0.91 (0.90,0.92) | 0.86 (0.84,0.87) |
|  | Recurred (n=2,748) | - | - | - | 2.83 (2.69,3) | 0.63 (0.61,0.65) | 0.30 (0.29,0.32) |
| **Adjuvant treatment subgroup** | Received carboplatin and paclitaxel with or without radiation (n=2,685) | 4.26 (3.59,4.97) | 0.62 (0.61,0.64) | 0.48 (0.46,0.50) | Not reached (8.96,NA) | 0.80 (0.78,0.82) | 0.61 (0.59,0.63) |
|  | dMMR & received carboplatin and paclitaxel with or without radiation (n=56) | 2.91 (2.2,NA) | 0.62 (0.51,0.77) | 0.50 (0.37,0.67) | Not reached (NA,NA) | 0.93 (0.86,1.00) | 0.89 (0.81,0.98) |

*Abbreviations: ECOG = Eastern Clinical Oncology Group, FIGO = International Federation of Gynecology and Obstetrics, MMR = Mismatch Repair, dmmr= Mismatch RepairDeficient.*

**Table S6: Aalen-Johnsen estimates of probabilities of first disease recurrence and death, respectively, from date of initiation of adjuvant therapy, as well as probabilities of death from disease recurrence**

|  | **Adjuvant therapy to Recurrence** | **Adjuvant therapy to Death** | **Recurrence to Death** |
| --- | --- | --- | --- |
| **Time**  (Months) | Estimated Transition Probability (SE) (LCI-UCI) | Estimated Transition Probability (SE) (LCI-UCI) | Estimated Transition Probability (SE) (LCI-UCI) |
| **6** | 0.14 (<0.01) (0.13- 0.15) | <0.01 (<0.01) (<0.01- <0.01) | <0.01 (<0.01) (<0.01- <0.01) |
| **12** | 0.25 (0.01) (0.24- 0.26) | <0.01 (<0.01) (<0.01- <0.01) | <0.01 (<0.01) (<0.01- 0.01) |
| **18** | 0.31 (0.01) (0.29- 0.32) | 0.04 (<0.01) (0.04- 0.05) | 0.05 (0.01) (0.03- 0.06) |
| **24** | 0.33 (0.01) (0.32- 0.35) | 0.11 (<0.01) (0.1- 0.12) | 0.12 (0.01) (0.1- 0.14) |
| **36** | 0.33 (0.01) (0.31- 0.34) | 0.23 (0.01) (0.22- 0.24) | 0.26 (0.01) (0.24- 0.29) |
| **60** | 0.25 (0.01) (0.23- 0.26) | 0.46 (0.01) (0.45- 0.48) | 0.5 (0.02) (0.47- 0.54) |

*Abbreviations: LCI = Lower confidence interval, UCI= Upper confidence interval, SE= standard error.*

*
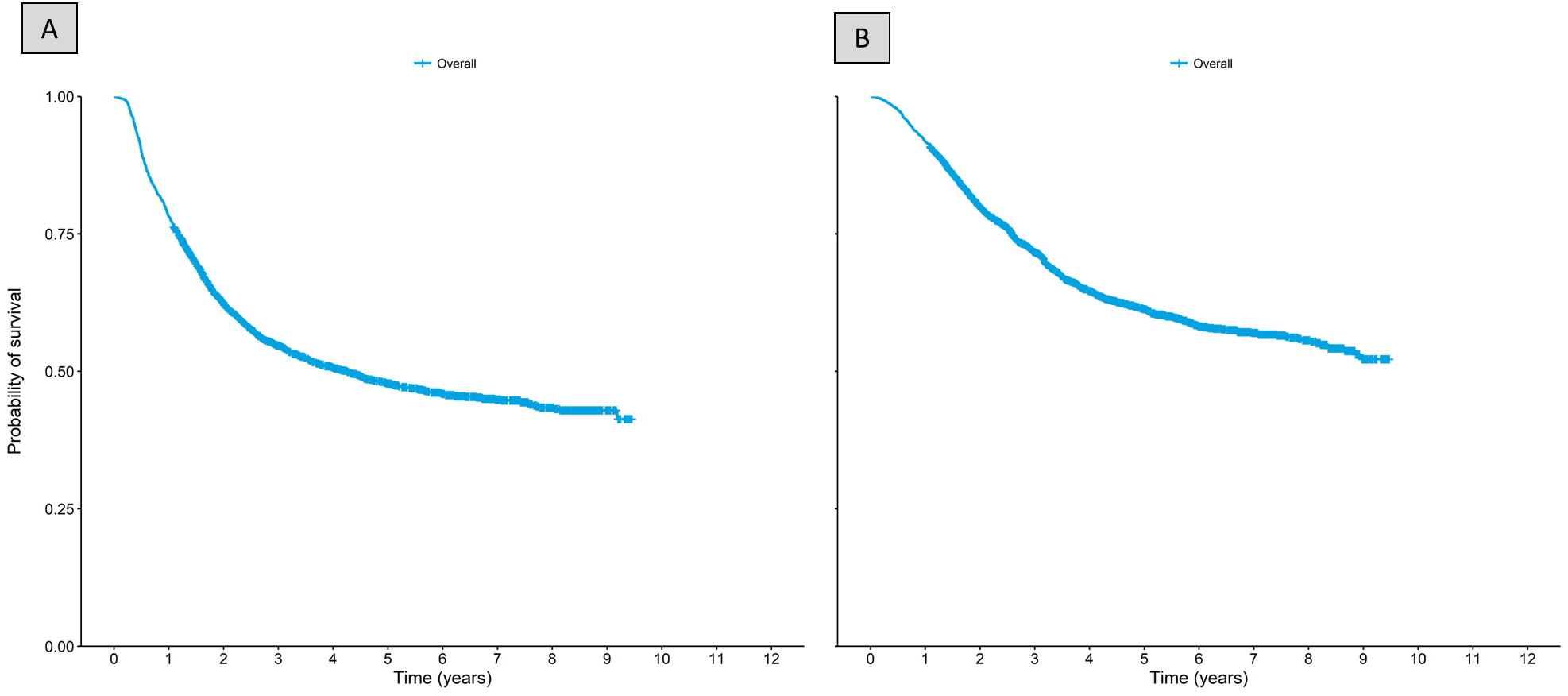
*

**Figure S1: (A) Real-world disease-free survival and (B) Overall survival from initiation of first adjuvant therapy, restricted to patients receiving carboplatin + paclitaxel +/-RT as adjuvant therapy**


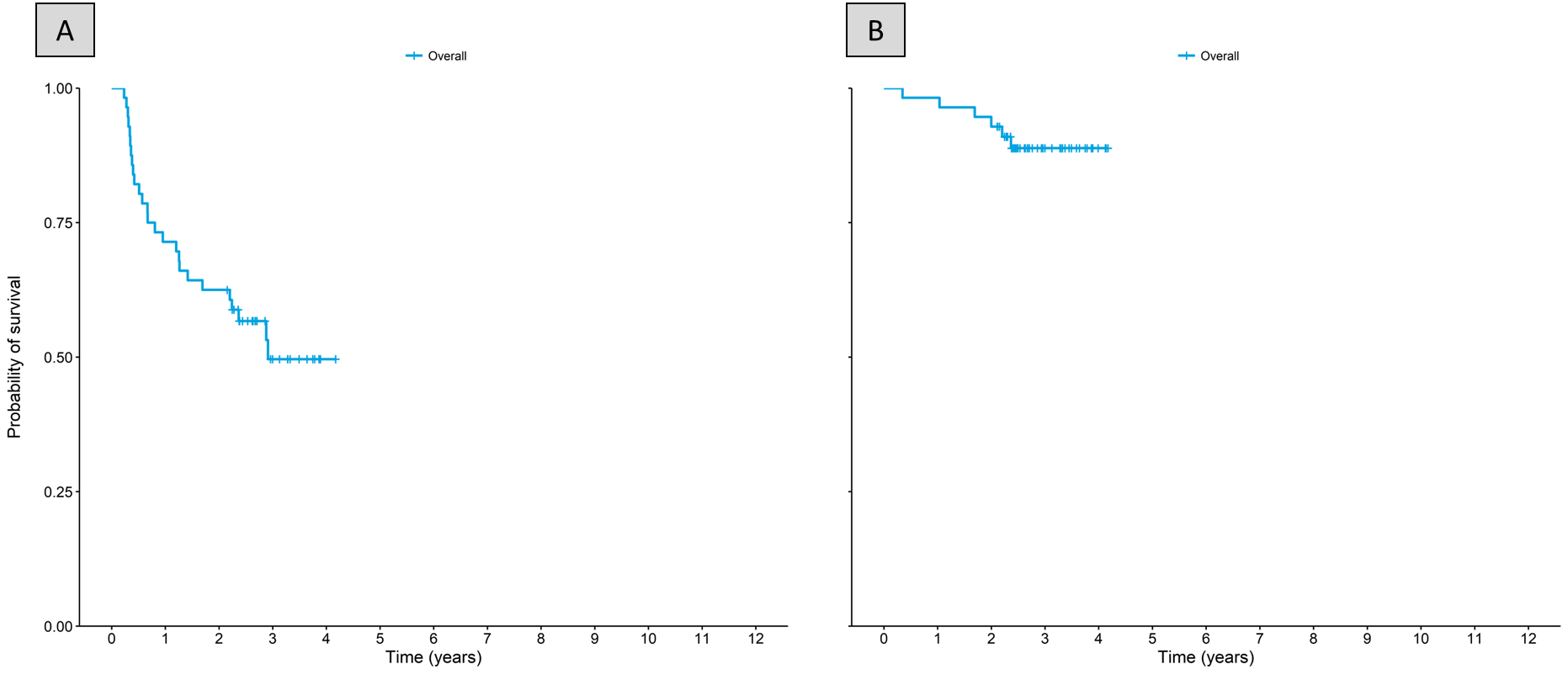


**Figure S2: (A) Real-world disease-free survival and (B) Overall survival from initiation of first adjuvant therapy, restricted to patients receiving carboplatin + paclitaxel +/-RT as adjuvant therapy and dMMR**
